# Litholapaxy in the Male under Local Anesthesia

**Published:** 1897-09

**Authors:** James Pedersen

**Affiliations:** Instructor in venereal and genito-urinary diseases, New York Post-Graduate Medical School and Hospital


### LITHOLAPAXY IN THE MALE UNDER LOCAL ANESTHESIA.

By JAMES PEDERSEN, M. D.,
Instructor in venereal and genito-urinary diseases, New York Post-Graduate Medical School and Hospital.

LOCAL anesthesia by cocaine or eucaine for the performing of this operation may justly be considered a step in advance of the more usual employment of general anesthesia by ether or chloroform. As it secures for the patient a saving of time and a minimum of risk, we should endeavor to apply this improved technique whenever possible.

Not every case is suited to litholapaxy under merely local anesthesia. A small or even medium-sized calculus, be it hard or soft, in a not over-irritable adult bladder, without prostatic hypertrophy, is the ideal condition. In proportion as this is departed from in one or more of its factors, the operation grows in difficulty, until finally we are met by positive contra-indications. These may be : (1) A large calculus ; (2) a bladder so irritable that it will not retain a volume of fluid sufficient to make possible and safe the necessary excursions of the lithotrite during the manipulations ; (3) partially obstructive prostatic hypertrophy, making the introduction of the lithotrite—still more so of the evacuator—difficult and painful by reason of the unavoidable pressure brought to bear upon the gland-mass ; (4) a post-prostatic pouch in which a calculus or fragments cannot easily be reached with the tip of the lithotrite ; (5) a badly sacculated bladder ; (6) a patient so exhausted by protracted suffering that he cannot endure even that degree of discomfort and pain which cannot be avoided. Finally, litholapaxy without general anesthesia should not be undertaken in children and very young subjects.

From the foregoing it is manifest that a knowledge, on the part of the surgeon, of the patient, his prostate, bladder and its contents, gained by careful personal examination, is a necessary prerequisite to a decision in favor of local anesthesia, and also that the surgeon should have the necessary experience and dexterity, and a "fine adjustment" of the tactile sense. In point of skill required, litholapaxy under local anesthesia is to litholapaxy under general anesthesia what the latter is to suprapubic lithotomy.

The method of procedure is as follows: the patient should lie flat on his back with his hips slightly elevated. The thigh and legs may be extended on the same level or be slightly flexed, or the legs may hang down over the edge of the table. After gentle irrigation of the bladder with a warm boracic acid solution, inject into the bladder through the catheter ℥ ij. of a four per cent. solution of cocaine or eucaine. The latter is now preferred, because of its lesser toxic effects. Withdraw the catheter. With a Bangs' syringe-sound, of a size which will enter the meatus without stretching it, instill *M* xx. to xxx. of the same anesthetising solution into the prostatic and membranous portions of the urethra, and leave the instrument with its beak resting at the bulbo-membranous junction to help retain the solution thus instilled. At the end of six or seven minutes anesthesia will have been obtained. Remove the syringe-sound, re-introduce the catheter, evacuate the solution in the bladder, inject ℥ v. to vi. of warm boracic acid solution, and withdraw the catheter. Thus far the lubricant used must be some material soluble in water, for example, sterilised glycerine. From now on a lubricant with more "body," such as sterilised vaseline, may be used, and the crushing of the calculus and evacuation of the fragments and débris is proceeded with in the ordinary way.

If cocaine, instead of eucaine, be employed, the condition of the bladder-wall should be taken into account, and the patient's general condition watched. A diseased bladder mucous-membrane (as in chronic cystitis) more readily absorbs than does one covered with healthy epithelium.

As litholapaxy under local anesthesia is usually done to remove a small calculus, a soft, secondary calculus, or a fragment accidentally or unavoidably left behind after some previous operation, Chismore's evacuating lithotrite is of special advantage, because of the rapidity and certainty possible with it and because of the consequent economy in manipulations. The value of the latter at once becomes apparent when we recollect that the unavoidable distress felt by the patient is a deep-seated pain, due to pressure upon the prostrate body when the lithotrite is being moved this way and that in the initial search for the calculus or fragment.

If, at the moment of introducing the lithotrite, the meatus proves too small to admit it, a few minims of the four per cent. solution can be injected and the meatus cut. If desired, the whole penile urethra may be anesthetised also.

In many cases, notably those presenting a fragment or a small secondary calculus, the operation of litholapaxy under local anesthesia can be done in the surgeon's office and the patient allowed to go about his duties after resting a few minutes. This is an advantage which he greatly values. In other cases, however, there is a greater advantage to be gained by having the patient remain in bed a few hours preceding and following the operation.

29 East 44th Street. —*Post-Graduate*, August.

---

A New Dressing.—In *Cincinnati Lancet-Clinic*, Dr. Charles A. L. Reed, of Cincinnati, at the recent meeting of the Cincinnati Obstetrical Society, described before that body a new dressing with which he had been made familiar during a visit to Mexico. This new dressing is asbestos, prepared in various lengths and widths, and it can be put to the various uses for which ordinary surgical dressings are employed. For instance, in the matter of sponges, asbestos being perfectly pliable, eminently absorptive, smooth and soft, it is well adapted to the purposes of a sponge without possessing any of the harshness or roughness of sponges made from other material. Dr. Reed states that it can be made into wicks for the purpose of uterine drainage, although he questions its usefulness here owing to its delicate texture, such frailty rendering it prone to breakage during attempt at withdrawal. But the best recommendation consists in the certainty of its sterilisation. Placing the asbestos upon a sterile plate, adding a small quantity of alcohol and lighting, complete sterilisation is assured.—*Northwestern Medical and Surgical Reporter*.

---

To Differentiate between a tumor of the right kidney and a tumor of the liver, inflate the rectum and colon. If the tumor is of the kidney the inflated intestines will lie in front of it, and there will be a tympanitic resonance; if the tumor is of the liver, it is rendered more prominent and dulness occurs on percussion.—*Pract. Med.*

